# Hypoxia within subcutaneously implanted macroencapsulation devices limits the viability and functionality of densely loaded islets

**DOI:** 10.3389/frtra.2023.1257029

**Published:** 2023-11-17

**Authors:** Samuel A. Einstein, Leah V. Steyn, Bradley P. Weegman, Thomas M. Suszynski, Athanassios Sambanis, Timothy D. O’Brien, Efstathios S. Avgoustiniatos, Meri T. Firpo, Melanie L. Graham, Jody Janecek, Lynn E. Eberly, Michael Garwood, Charles W. Putnam, Klearchos K. Papas

**Affiliations:** ^1^ Center for Magnetic Resonance Research, Department of Radiology, University of Minnesota, Minneapolis, MN, United States; ^2^ Department of Radiology, The Pennsylvania State University, Hershey, PA, United States; ^3^ Department of Surgery, University of Arizona, Tucson, AZ, United States; ^4^ Sylvatica Biotech Inc., North Charleston, SC, United States; ^5^ Department of Plastic Surgery, University of Texas Southwestern Medical Center, Dallas, TX, United States; ^6^ Department of Chemical & Biomolecular Engineering, Georgia Institute of Technology, Atlanta, GA, United States; ^7^ Veterinary Population Medicine Department, University of Minnesota, Saint Paul, MN, United States; ^8^ Department of Medicine, Stem Cell Institute, University of Minnesota, Minneapolis, MN, United States; ^9^ Department of Surgery, Preclinical Research Center, University of Minnesota, Saint Paul, MN, United States; ^10^ Division of Biostatistics, University of Minnesota, Minneapolis, MN, United States

**Keywords:** islet transplantation, immunoisolation, fluorine-19, magnetic resonance spectroscopy, tissue engineering, hypoxia

## Abstract

**Introduction:**

Subcutaneous macroencapsulation devices circumvent disadvantages of intraportal islet therapy. However, a curative dose of islets within reasonably sized devices requires dense cell packing. We measured internal PO2 of implanted devices, mathematically modeled oxygen availability within devices and tested the predictions with implanted devices containing densely packed human islets.

**Methods:**

Partial pressure of oxygen (PO2) within implanted empty devices was measured by noninvasive ^19^F-MRS. A mathematical model was constructed, predicting internal PO2, viability and functionality of densely packed islets as a function of external PO2. Finally, viability was measured by oxygen consumption rate (OCR) in day 7 explants loaded at various islet densities.

**Results:**

In empty devices, PO2 was 12 mmHg or lower, despite successful external vascularization. Devices loaded with human islets implanted for 7 days, then explanted and assessed by OCR confirmed trends proffered by the model but viability was substantially lower than predicted. Co-localization of insulin and caspase-3 immunostaining suggested that apoptosis contributed to loss of beta cells.

**Discussion:**

Measured PO2 within empty devices declined during the first few days post-transplant then modestly increased with neovascularization around the device. Viability of islets is inversely related to islet density within devices.

## 1. Introduction

Islet replacement therapy remains a potential cure for type 1 diabetes (1–9). Despite recent improvements in islet transplantation, only about half of patients treated at the most experienced centers are insulin independent five years after transplantation (2–5, 10–19). The current standard for islet transplantation—the intraportal delivery of islets—is admittedly imperfect due to significant islet graft dysfunction and loss over time. Factors contributing to suboptimal engraftment in the liver include: blood-mediated reactions, resulting in inflammation and thrombus formation (20–23); recurrent autoimmunity (24); immunosuppressive drug-related cytotoxicity (25–33); and poor oxygenation (34–44). Importantly, cells transplanted into the liver are difficult to monitor for survival and function and are irretrievable (45–49). Because of the challenges and limitations imposed by intrahepatic transplantation of islets, there has been persistent interest in and a resurgence of investigational studies focused on developing extrahepatic tissue-engineered islet grafts, a “bioartificial pancreas” (50–55).

Although extrahepatic islet transplantation using tissue-engineered grafts (TEGs) obviates many of the limitations imposed by intraportal islet transplantation, the bioengineering, experimental, and clinical implementation of a TEG-based approach introduces its own challenges, including: (1) To avoid the necessity for systemic immunosuppression to protect allografted islets, the encapsulation strategy may incorporate an immunoisolation membrane; this prevents host immunomodulatory cells from gaining access to the islets. However, the unintended but unavoidable consequence of immunoisolation is to prevent host vascular penetration into the graft; biophysically, this distances the vasculature from the encapsulated islets, creating an additional hindrance to the rapid diffusion of gases and small molecules. The consequence is that delivery of oxygen and nutrients to the islets and the efflux of insulin and other effectors from the graft is to a degree impeded (50, 56–63). (2) Because transplantation of large numbers of islet equivalents (IE) based on body weight (>5,000 IE/kg) are needed to achieve insulin independence (64–66), grafts must be seeded at high densities in order to use devices of a reasonable size for patients (43, 57, 67–70). (3) The implantation site for the device is no less important; the optimal site would ensure proper access to nutrients and allow for efficient insulin secretion (59, 60). Many extrahepatic sites have been considered and investigated (38, 39, 47, 71–78) but the local partial pressures of oxygen (PO2) at these proposed sites is seldom reported, especially within TEGs. Adequate oxygenation is critical: the survival and especially the functionality of islets are highly sensitive to hypoxic or anoxic conditions (79–83). Accurate and precise monitoring of oxygenation status is therefore a critical parameter in the design and implementation of therapeutic TEGs.

This study investigated the effects of increasing islet density on the viability and function of TEGs implanted in either the subcutaneous space or the intraperitoneal cavity of inbred rats. Internal PO2 of sham (acellular) TEGs was measured using fluorine-19 magnetic resonance spectroscopy (^19^F-MRS); this technique has been validated for the measurement of TEG PO2 *in vitro* and *in vivo* (84, 85). These measurements, at intervals over 29 days post implantation, were employed to inform a mathematical model constructed to predict the effects of PO2 external to a device loaded with high islet densities, on graft viability and function. The model was then challenged by loading TEGs with islets at various densities, then implanting them in athymic nude rats (to minimize immunological reactivity) for one week. The devices were then explanted and the viability of their cellular contents measured by oxygen consumption rate (OCR); the data obtained were compared to predictions from the mathematical model.

## 2. Methods

### 2.1. Ethics statement and experimental schema

All animal research was performed with the approval of and in accordance with guidelines of the University of Minnesota and the University of Arizona Institutional Animal Care and Use Committees (IACUC). Procurement of human islets was approved and overseen by the University of California Institutional Review Board and informed consent was obtained for all donors.

**Figure 1:**
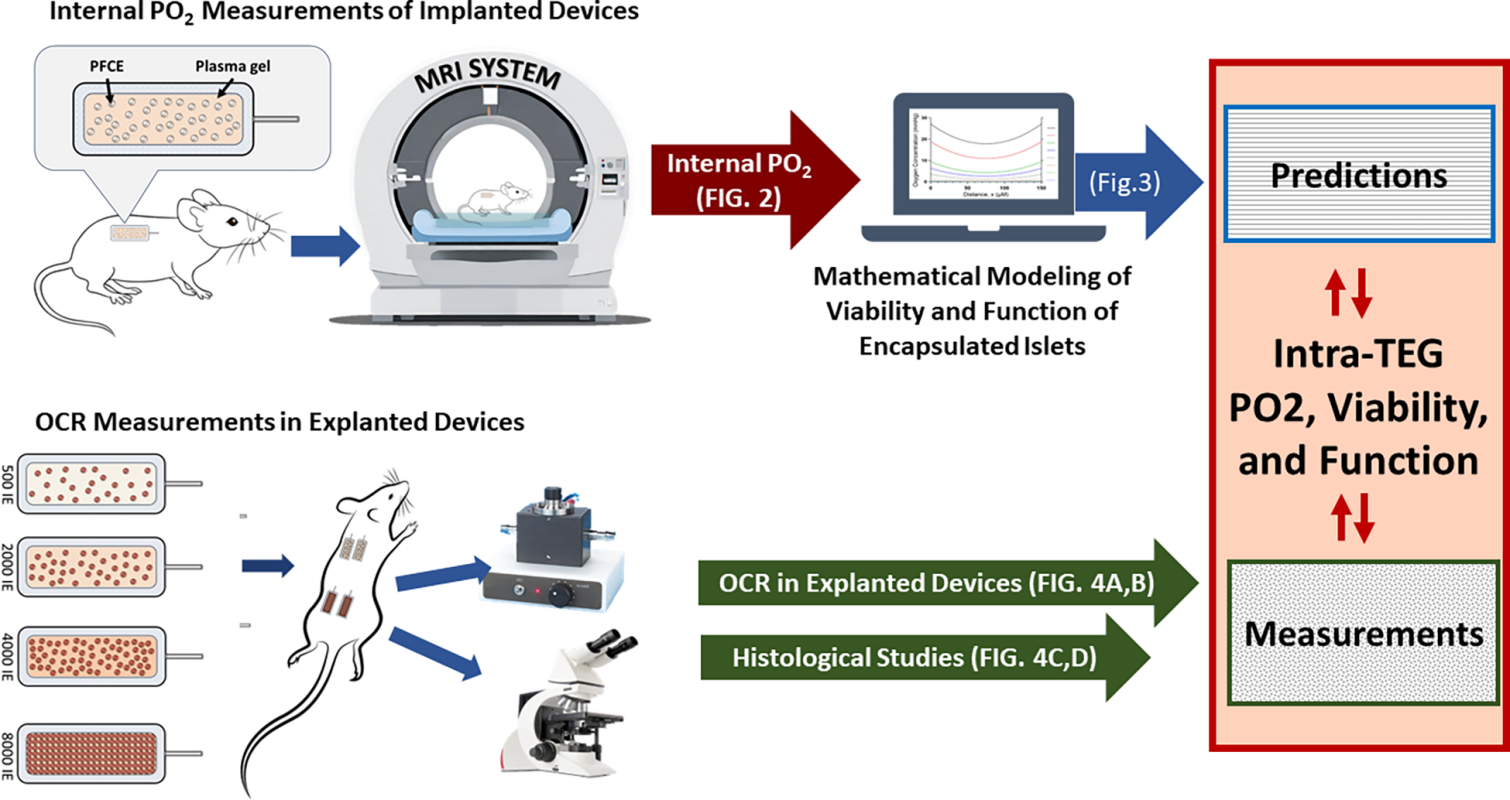
Schema for the experiments reported herein. Rats implanted with sham TEGs containing perfluoro-15-crown-5-ether (PFCE) and hydrogel, but without islets, were submitted to non-invasive fluorine-19 magnetic resonance spectroscopy (^19^F-MRS) to measure the partial pressure of oxygen within the *in vivo* devices. The data obtained informed the mathematical model constructed to estimate graft viability and function for various islet graft densities. The model results were challenged by implanting islet-loaded TEGs at different densities and measuring their viabilities by OCR after explantation. The OCR values obtained were used to calculate fractional viability and functionality, the final outputs. All implanted TEGs were examined histologically after their removal.

The experimental schema is described and illustrated in Figure 1.

### 2.2. Sham (acellular) tissue-engineered graft (TEG) construction

The contents of TEG constructs for ^19^F-MRS were constituted as a matrix and protected within a clinically-established macroencapsulating immunoisolation device (TheraCyte Inc., Laguna Hills, CA, USA) (59, 86–92), a device chosen for its flexibility, biocompatibility, and published record of successful pre-clinical (86, 93–98) and clinical implementation (91). All implantation procedures were performed using sterile techniques, materials, and reagents.

Sham implants (without islets, *n* = 9) were loaded with equal volumes of porcine plasma (Sigma Aldrich, St. Louis, MO, USA) and perfluoro-15-crown-5-ether (PFCE) (Exfluor Research Corporation, Round Rock, TX, USA), a type of perfluorocarbon with high oxygen solubility. The emulsion was injected into a 40 *μ*l immunoisolation device using a 250 µl precision syringe (Hamilton Company, Reno, NV, USA), then cross-linked with 5% v/v bovine thrombin solution. The latter was prepared by diluting concentrated topical thrombin solution (GenTrac Inc., Middleton, WI, USA) in phosphate-buffered saline with calcium and magnesium. After loading the TEG, the cell access port was trimmed and sealed with adhesive (Dermabond, Ethicon Inc., Somerville, NJ, USA).

### 2.3. Surgical implantation of sham TEGs

To measure the oxygenation status of TEGs *in vivo*, individual sham TEGs containing PFCE (see 2.2) were implanted in the subcutaneous space (*n* = 6) or the peritoneal cavity (*n* = 3) of non-diabetic Lewis rats (RT1^I^, Charles River Laboratories International, Inc., Wilmington, MA, USA). Anesthesia was induced with isoflurane by inhalation and maintained by spontaneous ventilation of isoflurane (1%–3%). The surgical site was clipped of hair and the skin prepped with chlorhexidine or an equivalent antiseptic. For subcutaneous implants, a 1.5 cm dorsal incision was made just inferior to the scapulae and perpendicular to and symmetrical about the mid-line. Using gentle blunt dissection, a small pocket sufficient to accommodate the device was created. After rinsing the pocket with saline, the device was placed in the pocket. For peritoneal implants, a 1.5 cm incision was made through the anterior abdominal wall to expose the peritoneum. The TEG implant was gently introduced into the abdominal cavity and tacked to the peritoneum using non-absorbable sutures, taking care not to place the TEG directly beneath the incision. The abdominal fascia and skin were closed with absorbable suture and the incision sealed with surgical glue (Dermabond). For postoperative analgesia, a nonsteroidal anti-inflammatory drug (NSAID) (meloxicam 1 mg/kg) was administered by subcutaneous injection once daily for at least 3 days. Animals were monitored daily until the incision was fully healed. After completion of the study, anesthetized animals were euthanized by inhalation of 100% carbon dioxide.

### 2.4. ^19^F-MRS oximetry

**Figure 2:**
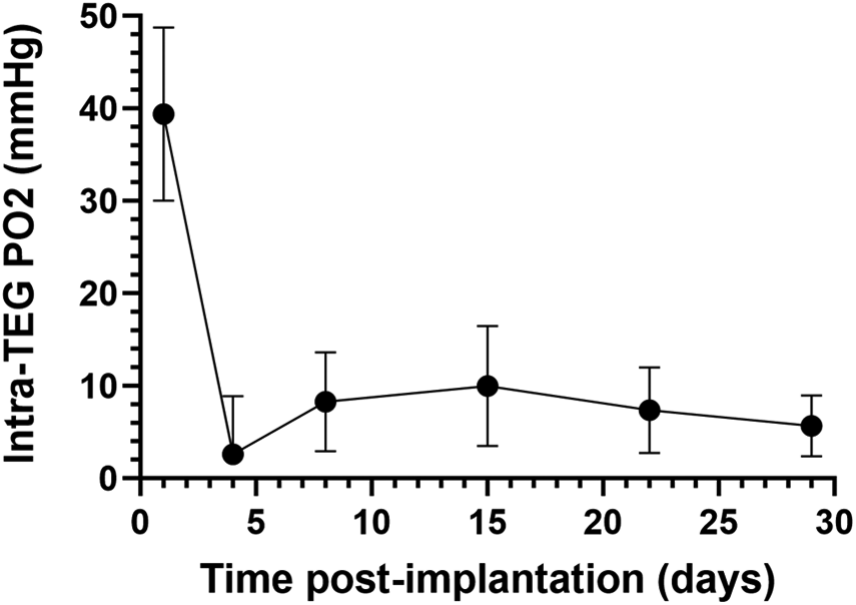
Oxygen partial pressure (PO2) measurements in subcutaneously implanted sham TEGs, as measured with non-invasive fluorine-19 magnetic resonance spectroscopy (^19^F-MRS). Average PO2 of subcutaneously implanted sham TEGs (*N* = 6) were measured on days 1, 4, 8, 15, 22, and 29 post-implantation. The mean PO2 was significantly higher (*p* < 0.0001) on day 1 than any subsequent time point, but by day 4 the devices were profoundly hypoxic. Similar trends were observed at the intraperitoneal site (Supplemental Information Figures S1A and B).

Oxygen measurements from implanted (see 2.3) TEGs containing PFCE (see 2.2) were acquired with a 16.4 tesla, horizontal-bore MRI system (Agilent Technologies, Santa Clara, CA, USA), Figure 1. Anesthesia was induced and maintained with inhaled isoflurane (1%–3%) in 47%–49% oxygen; the slight variations in oxygen content occurred as isoflurane flow was adjusted to maintain appropriate sedation. The rat was immobilized using a holder which centered the TEG over a 1.5 cm radius, custom-built, single-loop surface coil tuned to the ^19^F resonance frequency (656.8 MHz). The holder was inserted into the MRI system for scanning, during which the body temperature of the rat was carefully stabilized at 37 ± 0.2°C, as measured with a rectal thermocouple and regulated with a forced-air heater. The spin-lattice relaxation rate constant (R_1_) was measured using an inversion-recovery pulse sequence with adiabatic pulses. The inversion-recovery curve was fitted to the Bloch equation solution for longitudinal magnetization, using 3-parameter non-linear regression, with Graphpad-Prism software (Graphpad Software Inc., La Jolla, CA, USA). Each R_1_ was measured in six replicates separated by 6 s intervals, to ensure complete relaxation between repetitions. The R_1_ of each measurement was converted to PO2 using the previously determined multiparametric calibration (84). *In vivo* PO2 was measured on days 1, 4, 8, 15, 22, and 29 postimplantation (Figure 2). It is important to note that *in vivo* PO2 values determined by ^19^F-MRS oximetry represent average measurements across each device (84).

### 2.5. Histology

Sham TEGs were explanted from Lewis rats after 29 days, fixed in 10% buffered formalin for ≥24 h, transferred to 70% ethanol solution, and then embedded in paraffin wax for histology. Three 5 µm sections from separate portions of each explant were examined by an experienced pathologist to assess the degree of vascularization, the presence of foreign body reaction (FBR), and the extent of fibrosis in the adherent tissue surrounding the TEG. The degree of vascularization in the tissue surrounding each TEG was scored in blinded fashion on a scale from 0 to 3, where 0 = *avascular;* 1 = *minimal vascularity*; 2 = *moderate vascularity*; 3 = *extensive vascularity*. FBR was scored by examining the extent of inflammatory cell infiltrate composed of multinucleated inflammatory giant cells and macrophages but absent of lymphocytes and plasma cells; the extent was scored: 0 = *none;* 1 = *minimal;* 2 = *mild*; 3 = *mild/moderate;* 4 = *moderate;* 5 = *moderate/severe;* and 6 = *severe.* The degree of fibrosis was scored on the same scale as FBR by examining the density of fibrosis in the surrounding tissue.

### 2.6. Mathematical modeling of viability and functionality of densely loaded islets in TEGs

**Figure 3:**
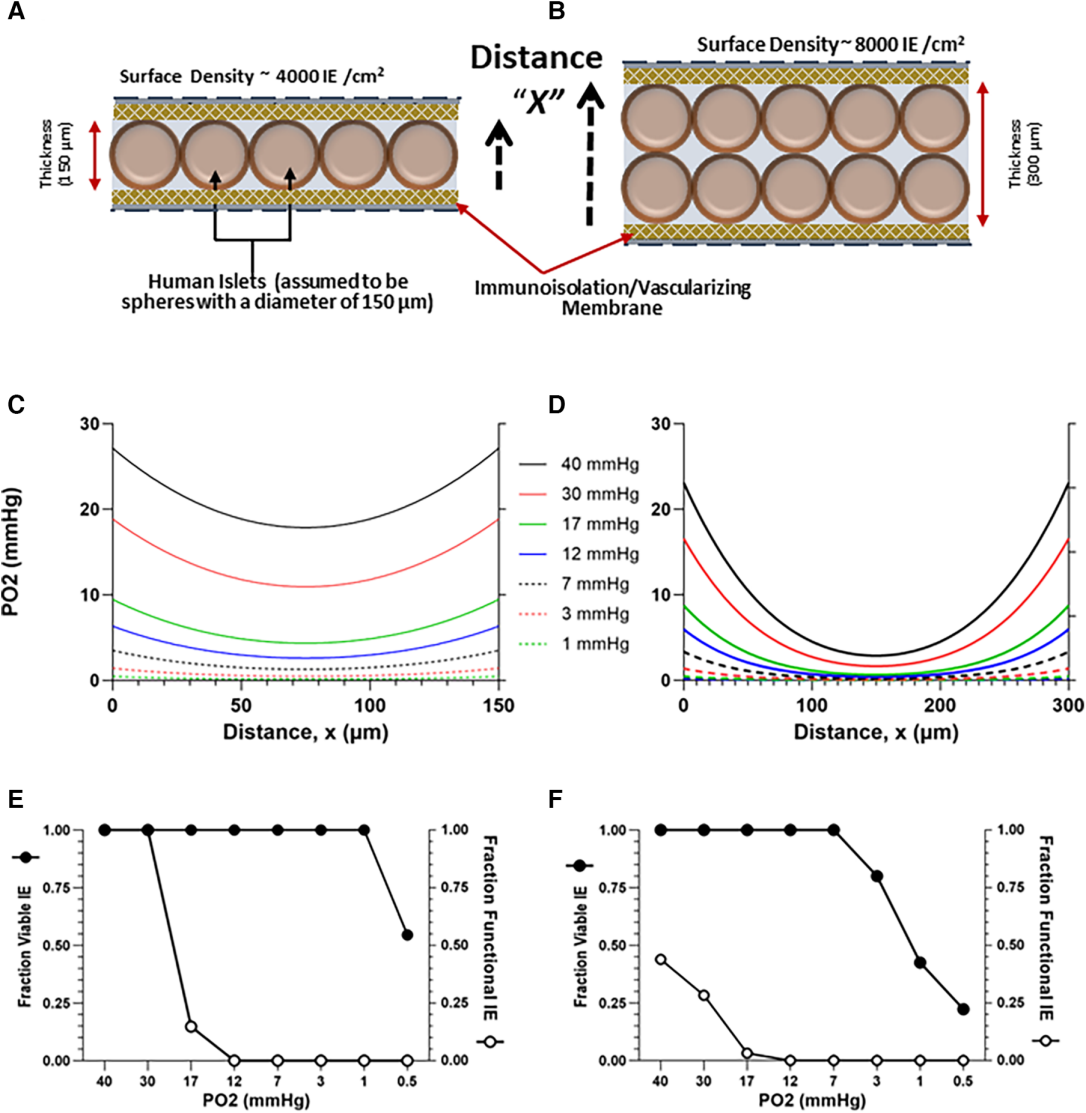
Model-estimated (see 1.6) oxygen profiles (**C,D**) and predicted viability and functionality (**E,F**) of human islets contained within TEGs at a planar-surface density of 4,000 or 8,000 IE/cm^2^, assuming a homogenous slab of either of two thicknesses, 150 (**A**) and 300 (**B**) µM and external ambient PO2s ranging from 0.5 to 40 mmHg; the distance X plotted on the x-axis in (**C,D**) corresponds to the dashed arrows in (**A,B**). Thirty mmHg is a value commonly assumed in the literature and 10 mmHg is a value representative of the steady state derived from *in vivo* ^19^F-MRS measurements (Figure 2). (**E,F**) Islet viability (threshold > 0.1 mmHg) and functionality (decreasing below 8 mmHg) are plotted against ambient PO2.

To elucidate the impact of external PO2 and cell density on TEG-contained islet viability and function, a diffusion-reaction oxygen transport model was developed, based on the size, shape, and dimensions of the devices employed in this study, using methods previously described (50, 63, 99–102). The dimensions for the TheraCyte devices used in the study are: 4.5 µl (17.5 mm × 7.0 mm × 2 mm) and 20.0 µl (22 mm × 11.2 mm × 3 mm). COMSOL Multiphysics software (COMSOL Inc., Stockholm, Sweden) was employed to develop a finite-element model describing the steady state, 1D diffusion-reaction condition, within a macroencapsulating TEG device. The TEG is modeled as a thin slab of oxygen-consuming tissue surrounded by the device membranes (Figures 3A,B). Oxygen diffusion into the device is described by the PO2 gradient between the exterior and interior of the implant and the permeability of the membrane (103). Transport of oxygen through the oxygen-consuming islet tissue is governed by two factors: diffusion through the tissue and oxygen consumption rate (OCR) of the tissue (63); an OCR of functioning, viable islets of 300 nmol/min/mg DNA (104) was used for this calculation. The model predicts the steady-state spatial oxygen concentration within the tissue content of TEGs containing densely packed islets (Figures 3C,D); from these oxygen concentrations, the fractional viability and functionality of the tissue within the central compartment of the device can be estimated (Figures 3E,F). We modeled a range of PO2 from 1 to 40 mmHg: a PO2 of 30 mmHg has been reported for *in vivo* tissues (103, 105–110); an external PO2 of 10 mmHg is congruent with the PO2 recorded within the sham TEG by ^19^F-MRS, see 2.4 and 3.1. Islets were considered to have some impairment of function at a steady-state PO2 of 8 mmHg and increasing loss of function as the PO2 diminished further. The islets were deemed non-viable when the steady-state PO2 was ≤0.1 mmHg (111). The parameters, equations and results of the simulations for islet cells are given in detail in the Supplementary Information File 1, and in Figure 3.

### 2.7. Explant OCR measurements

To test the effects of islet density on viability *in vivo* within TEGs, implants containing various quantities of human islets were implanted in rats for 7 days and then explanted for OCR measurements (Figure 1). TEGs (*n* = 19) were of the same construction as the sham devices described above (see 2.2), except that they were loaded with islets. Human islets were isolated at the University of California, San Francisco, cultured at 22**°**C in supplemented CMRL culture medium for up to 14 days, and shipped to the University of Arizona in 10 cm^2^ G-Rex devices (Wilson Wolf Manufacturing, New Brighton, MN, USA) (112, 113). Islets were quantified by DNA content (114–116) and aliquoted into tubes in various doses ranging from 500 to 8,000 IE per device. Each aliquot was then allowed to settle by gravity in its tube, the supernatant was removed, and the islets were re-suspended in 5 or 20 µl of sterile 1% sodium alginate solution. The islet suspension was then injected into the cell compartment of a 4.5 or 20 µl immunoisolation device, respectively, using a 100 µl precision syringe (Hamilton Company). The TEG was submerged in a 1.2 mM calcium chloride solution (PBS++) for 20–30 min to cross-link the alginate, then placed in PBS++ solution in preparation for implantation.

TEGs prepared with various doses of human islets were implanted in the subcutaneous space of 7 non-diabetic nude rats (athymic nude mutant, Hsd:RH-*Foxn1^rnu^*, Harlan Laboratories, Inc, Indianapolis, IN, USA). Each rat received two to four devices, each separately placed in its own subcutaneous pocket. Seven days later, the TEGs were surgically removed for measurements of OCR to assess islet viability and for immunohistochemical staining. The surgical implantation and explantation procedures were identical to those described for sham TEGs (see 2.3).

**Figure 4:**
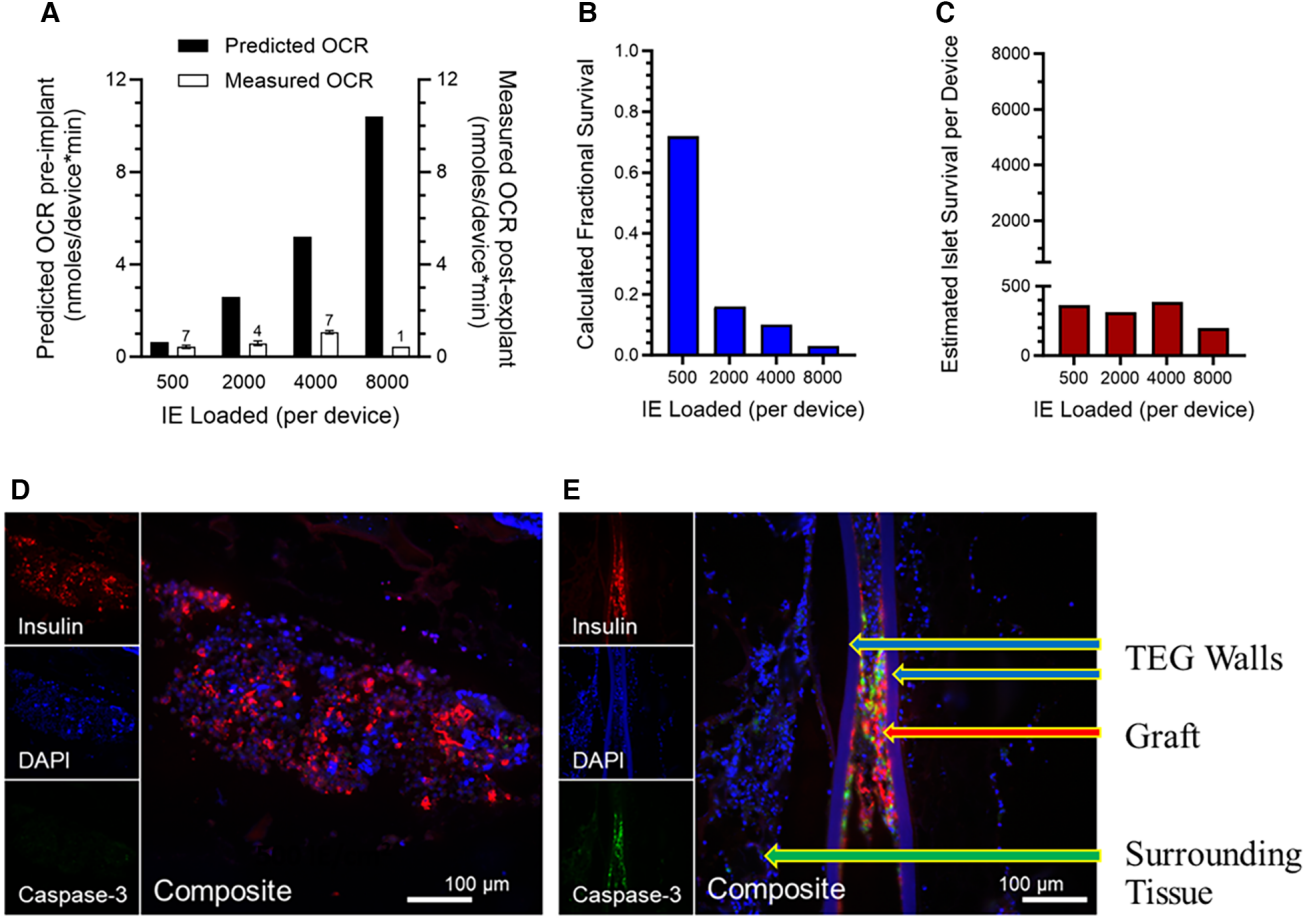
(**A**) Viability, measured by OCR of islets in explanted TEGs (*n* is given above the 'Measured OCR' columns), originally loaded with various densities and implanted in the SC space seven days previously; a dramatic decline in measured OCR in comparison to OCR predicted from the loading densities is observed with increasing islet density, far greater than that predicted by the mathematical model. (**B**) Fractional survival declined precipitously with increasing IE loading. (**C**) The absolute numbers of IE surviving number about 400 per device, regardless of the loading density. (**D,E**) Representative sections of the human islet cells within the same TEGs explanted after 7 days’ residence in the subcutaneous space of nude rats. The tissues are stained with the nuclear stain DAPI (blue) or by immunohistochemistry for either the insulin (red) or caspase-3 (green) proteins (see 2.8 and 3.5). Two densities of human islets within TEGs are illustrated: (**D**) low density, 500 IE/cm^2^, and (**E**) high density, 4,000 IE/cm^2^. At the greater density, there are fewer viable cells (DAPI), less production of insulin, and prominent caspase-3 protein. The composite image at low density demonstrates viable islet cells producing insulin and devoid of caspase-3 whereas at the higher density caspase-3 co-localizes with the remaining insulin-producing cells, suggesting that apoptosis and necrosis of the islet cells have occurred. Labeled are the walls of the TEG, the islet cell graft within it, and the surrounding tissue.

The OCR of each explanted TEG was measured by a method similar to that described for the assessment of free islet viability (117). The surgically recovered TEGs were stripped of adherent surrounding tissue, placed in a modified OCR chamber, and filled with air-saturated cell culture medium. In the sealed chamber, the PO2 of the medium was continuously monitored until the values achieved a linear slope; the OCR was calculated from the slope. By normalizing the OCR to the number of IE originally loaded into each device, individual islet graft viability (OCR/IE) was calculated. Previous OCR data obtained *in vitro* of human islets in devices under ideal conditions were used to calculate the “predicted OCR” at different densities (Figure 4A).

### 2.8. Immunohistochemistry of human islets in seven-day TEGs

TEGs containing various densities of human islets were explanted from nude rats after seven days, fixed in 4% paraformaldehyde and embedded in paraffin. Sections of 6 µm thickness were stained with DAPI (1 µg/ml, Roche, Indianapolis, IN, USA) or for insulin (guinea pig anti-porcine insulin 1:500, Dako, Carpinteria, CA, USA), and for cleaved caspase-3 (rabbit anti-human caspase-3, 1:250, Cell Signaling Technology, Danvers, MA, USA); detection was with affinity-purified secondary antiserum conjugated to donkey anti-rabbit IgG and donkey anti-guinea pig IgG (Alexa Fluor 488 and Alex Fluor 594, Jackson ImmunoResearch Laboratories, West Grove, PA, USA), as previously described (118). Independent images for each fluorophore were acquired using a Leica DM5500 microscope system, and the images were processed using Image Pro Plus 6.3 software (Media Cybernetics, Silver Spring, MD, USA); composite images were colored and assembled (blue = *DAPI*; red = *insulin*; green = *caspase-3*).

### 2.9. Statistical analysis

Average values are reported as the mean value and the standard error of the mean (SEM). Least squares-weighted-means and error values are reported for PO2 measurements due to large variabilities observed with some of the PO2 measurements. Statistical comparisons were performed with Graphpad-Prism software or SAS analysis package (version 9.2; SAS Institute, Cary, NC, USA).

## 3. Results

### 3.1. In vivo PO2 in empty subcutaneous and intraperitoneal TEGs

An important first step in modeling islet function and viability in TEGs was to obtain accurate measurements of oxygen concentrations within devices devoid of cells (see Figure 1). For this purpose, sham (acellular) TEGs loaded only with PFCE (2.2) were implanted in the subcutaneous space (*n* = 6) or peritoneal cavity (*n* = 3) of nine rats (2.3), monitored for 29 days using ^19^F-MRS oximetry (2.4), then explanted for histologic examination (2.5). Internal PO2 measurements were successfully acquired in all animals at six time points (1, 4, 8, 15, 22, and 29 days) following implantation.

The average internal PO2 of subcutaneously implanted sham TEGs one day following implantation was 39 (SEM 9) mmHg (Figure 2), about that of venous blood (40 mmHg). The day 1 value however was the highest obtained; it was significantly greater (*p* < 0.0001) than all values at successive time points. By day 4, the PO2 had decreased nearly to zero and then increased but remained at very low levels (∼10 mmHg) for the remainder of the time course. The same downward trend of PO2 over time was observed in the smaller set of IP implanted TEGs (Supplemental Information Figure S1A,B). A comparison of the PO2 values at each site on days 1 and 4 (Supplemental Information Figure S1A) were not statistically significantly different nor were the total time averaged PO2 values presented in Figure S1B, 12 (SEM 5) and 8 (SEM 10) mmHg for subcutaneous and intraperitoneal TEGs, respectively. Again, the difference was not statistically significant. It should be emphasized that the PO2 measurements using 19F-MRS oximetry of sham devices represent holistic measures of the entire TEG compartment, i.e., average values not limited to the boundary layer or other presumed sub compartments (84). Together, these data document that for at least the first month after implantation, the steady state partial pressure of oxygen within empty TEGs is in fact quite low, about 10 mmHg, in both the subcutaneous and intraperitoneal sites.

### 3.2. Histology of explanted sham TEGs

The low partial pressures of oxygen recorded within TEGs (see 3.1, above) may be the consequence of low blood vessel density around the empty devices and/or the presence of other oxygen consuming cells on the device surface, limiting oxygen transfer to its interior. Note that devices *containing cells* are reported to have increased densities of blood vessels around them, presumably due to pro-vascularization factors released by the implanted cells (119–122).

Hematoxylin and eosin-stained tissues surrounding explanted (day 29) *empty implants* from the immunocompetent Lewis rats were graded for successful vascularization and adverse fibrotic and foreign body reactions. Successful vascularization—an average score of 2.3 on a scale of 0 to 3—was evident; it was accompanied by only mild to moderate amounts of fibrosis (average score of 2.3/6) and a similar degree of foreign body reaction, average score of 2.7/6. Importantly, these mild responses are not to be confused with the fibrous encapsulation accompanying the traditional foreign body response (123) and were not directed toward the vascularizing membrane nor did they interfere with neovascularization at the device interface (124). This observation is in marked contrast to the classic host FBR observed in the absence of an appropriate vascularization membrane (125) or in reaction to SC LiPc-PDMS solid oxygen probes which were “enveloped by a fibrous capsule” after 6 weeks (126). Examination of the internal compartments of the TEGs showed remnants of the fibrin scaffold in all explants. No inflammatory cell infiltration was observed within the internal chamber of the TEGs, evidence that the immunoisolation chambers had not been breached. Thus, it appeared that TEGs remained intact for and were moderately-well vascularized by 29 days; images of explanted TheraCyte TEGs after 28 days residence in the host are shown in the Supplementary Information Figure S2). We conclude that host fibrotic and foreign body reactions, although observed, were mild in comparison to vascularization.

### 3.3. Predictions from the mathematical model of TEG oxygenation

Projections from the diffusion-reaction model of *in vivo* TEG oxygenation constructed *in silico* (see 2.6) to quantify the effects of implant site PO2 and islet cell density on TEG viability and function are presented in Figure 3. Curves for various oxygen concentrations external to the device are shown for cell layers of 4,000 (Figure 3A**)** and 8,000 (Figure 3B) IEQ/mL, in devices measuring 150 or 300 µM thickness, respectively, Figures 3C,D; the higher PO2 values are commensurate with assumptions often made in the literature (see Introduction); the lower PO2 values are representative of the data from *in vivo* ^19^F-MRS oximetry, described earlier (see 3.1).

Viability [which has a threshold of 0.1 mmHg (111)] was not affected in the thinner layer model by decreasing the external oxygen concentration (Figure 3E), except at the very lowest oxygen concentration (0.5 mmHg). However, the proportion of viable cells decreased at 3 mmHg in the 300 µM thickness model (Figure 3F).

As anticipated, the model predicts a far more dramatic decline in graft function [which is known to be compromised at ∼8 mmHg (57, 127) or lower, see 2.6], a much higher threshold than viability. In the thinner cell slab model (150 µM), loss of function by about 85% is predicted at an external PO2 of 17 mmHg; at 12 mmHg, nonfunctionality of all islets is anticipated (Figure 3F). In the 300 µM model, nonfunction of the majority of cells is predicted even at a 40 mmHg oxygen concentration (Figure 3F). The mathematical model thus predicts that, for a PO2 of 30 mmHg (the upper limit of previously expected values) and a planar-surface density of 4,000 IE/cm^2^, functionality remains intact in a thinner device but is severely compromised in the thicker device. Importantly, at the measured oxygen content of empty TEGs, namely ∼10 mmHg (see 3.1), the predicted functionality is essentially null under the conditions of the model (Figures 3E,F).

### 3.4. Estimation of viability of explanted TEG islet grafts by oxygen consumption rate

In light of the low internal PO2 of∼10 mmHg measured in TEGs (see 3.1) despite histologic evidence of successful vascularization (see 3.2 and Supplemental Information Figure S2), and the consequent results from the mathematical model (see 3.3**)** of severe loss of functionality with a PO2 of 10 mmHg, we sought data challenging the predictions posited by the model.

For this purpose, 19 immunoisolation TEGs of identical design to the sham TEGs were loaded with various numbers (determined by DNA content) of human islets suspended in alginate, then implanted subcutaneously in 7 nondiabetic nude rats; each rat received 2–4 devices in separate locations (see 2.7 and Figure 1). Seven days after implantation, the TEGs were surgically removed, stripped of adherent tissue, and placed in an OCR chamber for measurements of oxygen consumption rate (see 2.7). OCR was normalized to the number of IE loaded into each device and the individual islet graft viability (OCR/IE) was calculated. Logistically, it was not possible to measure OCR prior to implantation because of the attendant risk of infection after implantation. Instead, we used prior data from a study of OCR *ex vivo* of human islets in similar devices under ideal conditions; from these data, the predicted OCR for each density was calculated (Figure 4A).

As the model (Figure 3) had predicted, the measured viability (% viable tissue) decreased with increasing islet density at a pronounced rate, especially at low and moderate densities (Figure 4A). The calculated fractional survival likewise decreased with increasing IE loads; it was less than 20% for all densities except the lowest (Figure 4B). The estimated islet (IE) survival was also calculated (IE loaded*fractional survival); it was below 400 IE/device, even when 8,000 IE were loaded (Figure 4C).

Thus, the mathematical model portrayed trends in functionality and viability with accuracy; importantly, however, measured viability—especially at higher densities—did not meet the predicted rates and fell more precipitously than predicted by the mathematical model. This is consistent with the values measured on day 4 post implant (Figure 2) being well below the 10 mmHg measured at steady state when neovascularization had presumably occurred. In other words, until functional blood vessels are created, the cells that surround the device may deplete oxygen, lowering the intra-device PO2 to near zero until newly formed, functional vessels begin transporting oxygen-bearing RBCs to the area.

### 3.5. Immunohistochemistry of insulin and caspase-3 in explanted TEGs loaded with islets

To better understand the fate of islet cells at higher densities in TEGs, the devices retrieved from nude rats at 7 days for the *ex vivo* OCR viability study described above (3.4) were studied with immunohistochemistry identifying insulin or the caspase-3 protein, a marker of apoptotic activity (see 2.8). Representative images from TEGs loaded with low-density (500 IE/cm^2^) or high-density (≥2,000 IE/cm^2^) human islets are shown in Figures 4D,E). At low density, the islet cells displayed staining for insulin and minimal if any caspase-3 protein (Figure 4D) whereas sections from high-density TEGs exhibited more caspase-3 protein, which co-localized with the residual staining of the insulin protein (Figure 4E). Consequently, we were able to confirm that human islets loaded at higher densities within subcutaneous TEG implants indeed quickly lose viability, at least in part through apoptotic mechanisms, as well as coincident and subsequent necrosis.

## 4. Discussion

The intent of this study was to determine whether immunoisolating tissue engineered grafts (TEGs) implanted subcutaneously or intraperitoneally in rats would be successfully vascularized; and, if so, would they have ambient partial pressures of oxygen (PO2) sufficient to support both viability and importantly, the functionality of islet grafts of various densities contained therein. We conclude that even modest densities of islets are not functionally sustainable within such implanted TEGs.

One corollary of this conclusion is that in order for islet grafts to survive and function within encapsulated devices such as TEGs, they must be loaded at very low cellular densities (128, 129) – an unacceptable solution since TEGs of a clinically useful size would not accommodate sufficient islets to reverse diabetes. An alternative, and far more appealing corollary, is that islet grafts can be sustained at high densities in reasonably sized devices, *if* the chambers of the devices are supplemented with oxygen at super-ambient partial pressures (79, 128, 130, 131). In immunoisolating or similar encapsulating TEGs, supplemental oxygen might well be a permanent requirement; nonimmunoisolating devices—having more porous membranes which allow ingrowth of neovasculature—might achieve the necessary internal oxygen content within weeks to months, thus allowing cessation of supplemental oxygen. Various laboratories have developed novel approaches for providing increased oxygenation to encapsulated islets (53, 70, 79, 132–136).

Our conclusion regarding the inadequacy of oxygenation of the TEG internal environment was based upon both *in vivo* experimental studies and *in silico* mathematical modelling of the devices (Figure 1). First, using ^19^F-MRS oximetry of subcutaneously or intraperitoneally implanted sham TEGs, devoid of cells but containing PFCE, an average available PO2 of 12 (SEM 5) mmHg was measured for the SC site and only 6.8 (SEM 10) mmHg in the peritoneal space, after just a few days *in vivo* (Figure 2 and Supplementary Information Figure S2). At equilibrium *in vivo*, these measurements within sham (empty) devices can perhaps best be interpreted to represent the PO2 surrounding the implanted device, that is, the expected limit of oxygen available for transport into the device. Recently reported PO2 measurements with a solid, totally implantable probe yielded roughly comparable values and somewhat similar trends for the native SC tissue and IP cavity (126). Importantly, the internal PO2 available to islets within the TEG would perforce be lower than these measured values due to oxygen consumption by the islets themselves, with the lowest oxygen concentrations in the center of the device. At higher planar-surface densities, the available PO2 would be considerably lower, perhaps effectively anoxic, because the oxygen consumption rate of islet cells further sharpens the oxygen gradient. Examples of this limitation are widely reported in the literature and have recently been reviewed (128). We emphasize that these unanticipated low values were not the consequence of excessive fibrosis or foreign body reaction, as was confirmed by histologic examination; the devices were in fact well vascularized (Supplementary Information Figure S2).

Because the *in vivo* oxygen levels of implanted sham TEGs were monitored for a 29-day period following implantation, the dynamics of the changing PO2 portrayed in Figure 2 and Supplementary Information Figure S1A invite scrutiny. The PO2 on the first day following implantation was significantly higher than that recorded at all future time-points, likely due to the continued presence of residual oxygen in the TEGs (introduced during the preparation of the device at ambient PO2∼150 mmHG) and the surrounding surgical site. Then, a dramatic decrease in PO2, reaching near-zero levels, was observed (day 4). This is likely attributable to the recruitment of highly metabolic inflammatory cells to tissue damage at the surgical site. These cells not only compete for available oxygen but might also affect the graft indirectly though paracrine signaling and the local secretion of inflammatory cytokines; these possible effects were not addressed in our study. From days 8 to 22, we note a gradual, modest increase in PO2 within the devices; we hypothesize that this is the consequence of neovascularization in the SC or IP tissues surrounding the implanted TEG, slightly increasing the available oxygen. For the remaining 22–29 days following implantation, the ambient oxygenation appears to achieve a steady state. However, this vascular formation is insufficient to alleviate the hypoxic condition inside the immunoisolated TEG, especially when high densities of islets are required.

Mathematical modeling based on a range of external PO2s encompassing both 30 mmHg (a previously accepted oxygen concentration within sham devices) and 10 mmHg (representing our *in vivo* measurements) produced viability curves which were calculated with a threshold of 0.1 mmHg; the curves indicate that viability is sustainable at a moderate (4,000 IEQ/cm^2^) planar-surface density. Functionality, however, poses a stark contrast to viability: with a ∼100-fold higher oxygen requirement than viability, functionality is likely already declining at 8 mmHg and is projected to be unacceptable at *any* islet density when modeling the 10 mmHg environment (Figures 4E,F). The mathematical model for predicting viability based on the PO2 available within TEGs is limited by its initial assumptions that vary depending on the specific environment *in vivo* (*e.g.,* factors beyond hypoxia and anoxia may contribute to graft loss) and are influenced by the characteristics of the particular islet preparations used for actual TEGs. Recognizing the limitations of mathematical modeling, experimental studies are required to evaluate any model's predictive reliability.

The dire predictions of the model were confirmed experimentally by measuring oxygen consumption rate (OCR), as a surrogate for viability, in freshly explanted TEGs after one week's residence subcutaneously (Figure 1). The results of OCR measurements (Figures 4A–C) supported the trend derived from the mathematical model, that viability decreases with increasing islet density, but the experimental observations indicated a more pronounced drop in viability at modest densities (500 IE/cm^2^) and a more profound loss of viability at high islet densities (>2,000 IE/cm^2^) than predicted (Figures 3E,F). Histologic examination was confirmatory; at a density of 4,000 IE/cm^2^ (Figure 4E) compared to 500 IE/cm^2^ (Figure 4D), far fewer cells stained positive for insulin; importantly, staining for caspase-3 indicated that many of the remaining islet cells were undergoing apoptosis. Although the images documented apoptosis, it must be assumed that necrosis had also occurred; several reports have noted that the two processes are often coincident, perhaps sequential (137–139). However, whether both apoptosis and necrosis are both primarily the consequence of hypoxia alone remains to be determined.

Our data and conclusions should not be taken as a blanket condemnation of this technology. Transplanting cells inside a TEG offers essential advantages (68, 70, 140, 141): it localizes the graft within the device, the cells are therefore more amenable to *in vivo* assessments and, if need be, can be retrieved. Previous efforts (142) to develop a tissue-engineered islet graft (or bioartificial pancreas) have struggled to achieve clinical translation (58, 61, 63, 143–145), but with recent developments in stem cell and xenogeneic islet technologies (87, 146–155) and the promise of scalable alternative *β*-cell sources (142, 151, 156, 157), there is a renewed interest in—and progress toward—developing a functional insulin-producing, cell-containing TEG for diabetes treatment (87, 158–161).

Previously published reports described the challenges of oxygenation for TEGs, especially in scenarios which require high cell-densities to achieve the therapeutic objective with a device of practical size (50, 55, 75, 162). The sham TEG measurements of available oxygen *in vivo* suggest that the subcutaneous space is profoundly hypoxic, with an average available PO2 of 12 (SEM 5) mmHg [and only 6.8 (SEM 10) mmHg in the peritoneal space].

These trends in oxygen levels are not statistically significant, but they are consistent with the pathophysiologic processes that follow a surgical insult and the implantation of foreign materials. TEGs implanted in the peritoneum had a similar PO2 trend and a similar hypoxic environment as subcutaneous TEGs during the 29-day period of observation (Supplementary Information Figure S1). The histological findings from explanted sham TEGs support this interpretation of the dynamic PO2 changes *in vivo* (Supplemental Information Figure S2). By 29 days, the surrounding tissue had developed a moderate amount of neovascularization; in comparison, only mild fibrosis and foreign body reaction were identified. These findings are consistent with previous reports using similar TEGs (75, 99, 103); fibrosis and foreign body reactions are not unexpected with these materials but can be effectively mitigated with induction of neovascularization aided by the proper selection of materials and membrane microarchitecture (125, 163–165). It should be emphasized that neovascularization of TEGs having vascularizing external membranes is – in fact – a rapid process. Padera and Colton (164) have carefully documented the time course of vascularization of a suitable membrane: vascularity at the interface “increased up to 10 days and remained at this level even at 329 days post-implantation”. Similar observations have been reported by others (88, 165, 166). Investigations of pro-angiogenic factors and other approaches to accelerate vascularization have recently been reviewed (167).

In summary, these results suggest that without oxygen supplementation, thin devices with high oxygen permeability are required to maintain viability of encapsulated tissues; even then, to ensure adequate islet function, only low islet densities can be loaded. To accommodate TEGs with low islet densities (≤500 IE/cm^2^), very large implants would be required to achieve the surface area needed for oxygenation; a device of the required size would be impractical for implantation in humans (57). In addition to the hypoxic stress that encapsulated islets face within these TEGs, there are other *in vivo* considerations that may further decrease or limit the viability and function of such grafts. Although these considerations are beyond the scope of this work, they may help explain the greater viability loss observed in our experimental measurements using OCR in comparison to the model results. Oxygenation is especially challenging when using high cell densities, which create significant oxygen transport limitations within the graft (50). These challenges are recognized in recent reviews (50, 55, 128). Our results confirm a critical need for improved oxygenation in macroencapsulated TEGs to support the high-cell densities needed for therapeutic applications. Many engineered tissues for the treatment of human disease will require developing complex tissues with high (near native) cell densities to provide therapeutic benefit. Oxygenation is a critical challenge facing the field of tissue engineering and especially for encapsulation approaches that do not allow complete re-vascularization of tissues.

## Data Availability

The original contributions presented in the study are included in the article; further inquiries can be directed to the corresponding author.
