# Supplementary material for: Hypoxia within subcutaneously implanted macroencapsulation devices limits the viability and functionality of densely loaded islets
Source: Front Transplant. 2023 Nov 17;2:1257029. doi: 10.3389/frtra.2023.1257029 (PMC11235299; doi:10.3389/frtra.2023.1257029)
Supplement: Supplementary file 1 [file Image1.pdf]

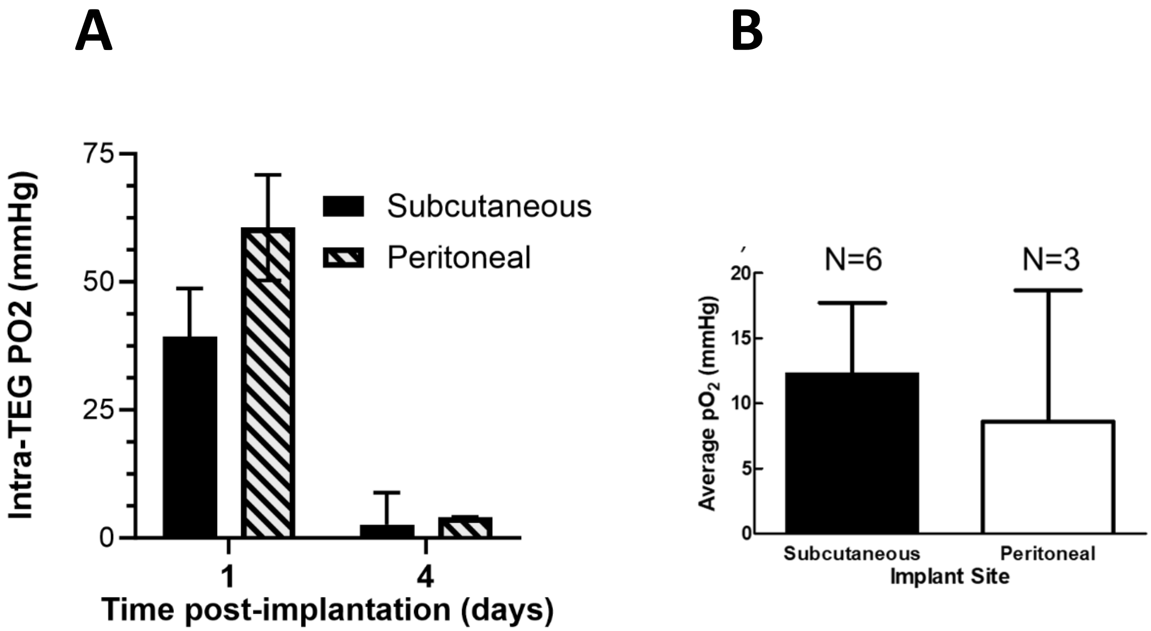

SUPPLEMENTAL FIGURE S1. Comparison of oxygen concentrations in devices implanted subcutaneously and intraperitoneally (SC data from **Figure 2**). **(A)** Comparison of the two device locations, SC and IP, on days 1 and 4; the differences are not statistically significant. **(B)** PO2 values averaged over time of SC and IP; again, the differences are not significant. Subcutaneous implantation, N = 6; intraperitoneal implantation, N = 3.
