# Supplementary material for: Hypoxia within subcutaneously implanted macroencapsulation devices limits the viability and functionality of densely loaded islets
Source: Front Transplant. 2023 Nov 17;2:1257029. doi: 10.3389/frtra.2023.1257029 (PMC11235299; doi:10.3389/frtra.2023.1257029)
Supplement: Supplementary file 2 [file Image2.pdf]

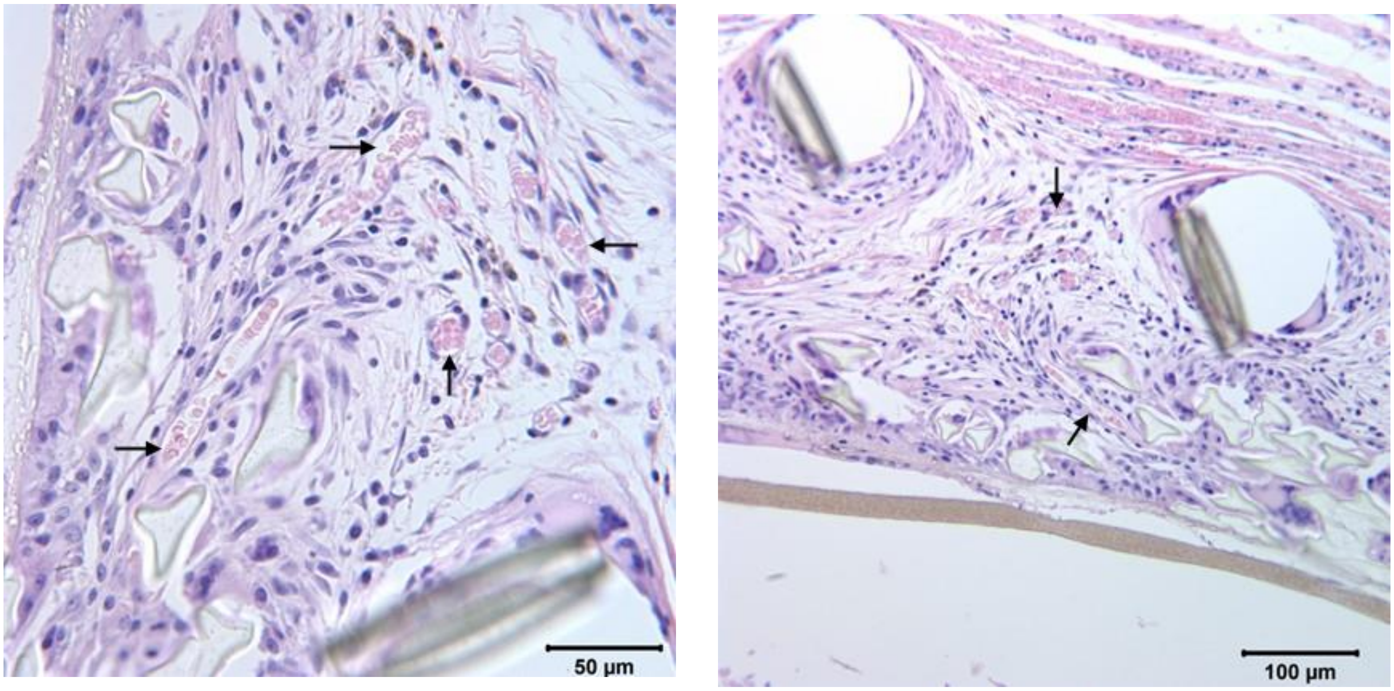

SUPPLEMENTAL FIGURE S2. Representative images of tissue surrounding the explanted TEG after 29 days *in vivo*. Vascular structures (black arrows) are visible in the tissue immediately surrounding and adherent to the vascularization membrane of the TEG. Red blood cells can also be seen inside the vascular structures newly formed in the remodeled tissue.
