# Supplementary material for: Hypoxia within subcutaneously implanted macroencapsulation devices limits the viability and functionality of densely loaded islets
Source: Front Transplant. 2023 Nov 17;2:1257029. doi: 10.3389/frtra.2023.1257029 (PMC11235299; doi:10.3389/frtra.2023.1257029)
Supplement: Supplementary file 3 [file Datasheet1.pdf]

Supplemental File 1. Mathematical Modeling: Equations and Parameters

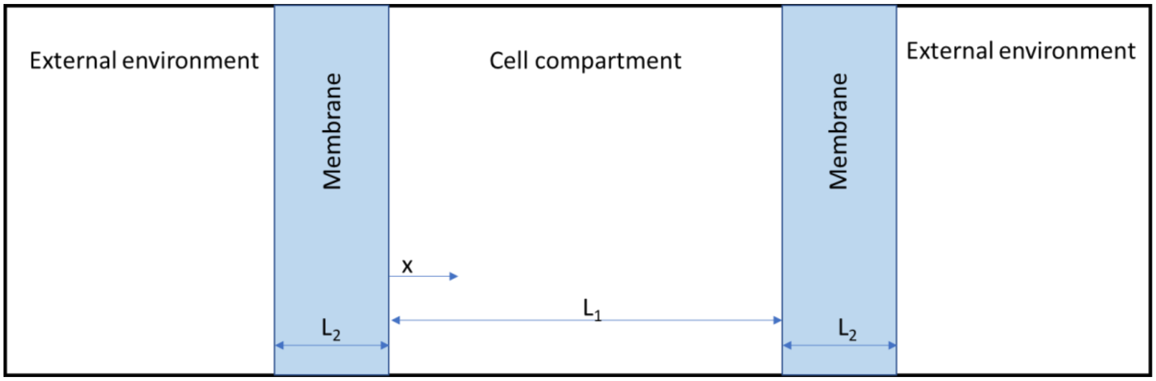

Consider a 1 cm x 1 cm x 150 μm construct. For islet equivalents (IEQs) of 150 μm diameter, the maximum number of IEQs in the construct is $(10^4/150)^2$ = 4444 IEQs or, approximately, 4000 IEQs.

Assume that each IEQ contains 10.4 ng of DNA. Therefore, the density in the construct is (4000/0.015) = $2.67\text{x}10^5$ IEQs/mL, or $2.77\text{x}10^6$ ng DNA/mL.

Mathematical Model

For simplicity, we assume a homogeneous cell compartment. This is a good approximation for 4000 IEQs per construct, which fill most of the construct volume. At steady state:

$$D\frac{d^2C}{dx^2} - \frac{VC}{K+C}N = 0$$

<u>Boundary conditions</u>

At x=0, $D_M \frac{\alpha C_B - \alpha C}{L_2} = -D\frac{dC}{dx}$

At $x = L_1/2$, $dC/dx = 0$

Symbols

N	cell density
L_1	thickness of cell compartment
L_2	thickness of each membrane
C	concentration of dissolved oxygen
C_B	concentration of dissolved oxygen in the surrounding medium
V, K	Monod model parameters for oxygen consumption by the cells
D	Diffusivity of dissolved oxygen in the cell compartment

$\alpha * D_M$ Partition coefficient of dissolved oxygen in membrane times diffusivity of dissolved oxygen in membrane (these parameters appear only as a product in the model)

Parameter values

$N = 2.77 \times 10^6$ ng DNA/mL

L_1=150 μm = 0.015 cm

L_2=20 μm = 0.002 cm

K= 1×10^{-5} mmol/mL

V=125 nmol oxygen/(mg DNA*min) =

2.08×10^{-12} mmol oxygen/(ng DNA*s)

D=9.9×10^{-6} cm^2/s (from Suszynski et al[1])

$\alpha * D_M$=3.89×10^{-6} cm^2/s (measured in the Papas lab for Biopore membranes, unpublished)

---

[1] T.M. Suszynski, E.S. Avgoustiniatos, K.K. Papas, Oxygenation of the Intraportally Transplanted Pancreatic Islet, J Diabetes Res, 2016 (2016) 7625947.
